# Target product profiles for digital health technologies including those with artificial intelligence: a systematic review

**DOI:** 10.3389/frhs.2025.1537016

**Published:** 2025-05-20

**Authors:** Trystan B. Macdonald, H. D. Jeffry Hogg, Jacqueline Dinnes, Lucy Verrinder, Gregory Maniatopoulos, Sian Taylor-Phillips, Bethany Shinkins, J. Kevin Dunbar, Ameenat Lola Solebo, Hannah Sutton, John Attwood, Michael Pogose, Rosalind Given-Wilson, Felix Greaves, Carl Macrae, Russell Pearson, Adnan Tufail, Xiaoxuan Liu, Alastair K. Denniston

**Affiliations:** ^1^ University Hospitals Birmingham NHSFT, Birmingham, United Kingdom; ^2^ Academic Unit of Ophthalmology, Institute of Inflammation and Aging, College of Medical and Dental Sciences, University of Birmingham, Birmingham, United Kingdom; ^3^ NIHR Birmingham Biomedical Research Centre, Birmingham, United Kingdom; ^4^ Department of Applied Health Research, School of Health Sciences, College of Medicine and Health, University of Birmingham, Birmingham, United Kingdom; ^5^ Biostatistics, Evidence Synthesis and Test Evaluation and Modelling, Institute of Applied Health Sciences, University of Birmingham, Birmingham, United Kingdom; ^6^ Lion Health, Stourbridge, United Kingdom; ^7^ The University of Leicester, Leicester, United Kingdom; ^8^ Warwick Screening, Warwick Medical School, University of Warwick, Coventry, United Kingdom; ^9^ Warwick Applied Health, University of Warwick, Coventry, United Kingdom; ^10^ Vaccination and Screening Directorate, NHS England, London, United Kingdom; ^11^ Moorfields Eye Hospital NHS Foundation Trust, London, United Kingdom; ^12^ Population Policy and Practice, UCL Great Ormond Street Institute of Child Health, London, United Kingdom; ^13^ Independent Researcher, Oxford, United Kingdom; ^14^ Alder Hey Children’s Hospital, Liverpool, United Kingdom; ^15^ Hardian Health, London, United Kingdom; ^16^ St. George’s University Hospitals NHSFT, London, United Kingdom; ^17^ Digital Policy Unit, Department of Health and Social Care, London, United Kingdom; ^18^ Faculty of Medicine, School of Public Health, Imperial College London, London, United Kingdom; ^19^ Nottingham University Business School, University of Nottingham, Nottingham, United Kingdom; ^20^ Care Quality Commission, London, United Kingdom; ^21^ Medicines and Healthcare Products Regulatory Agency, London, United Kingdom; ^22^ Institute of Ophthalmology, University College London, London, United Kingdom; ^23^ College of Medical and Dental Sciences, University of Birmingham, Birmingham, United Kingdom; ^24^ Birmingham Health Partners Centre for Regulatory Science and Innovation, Birmingham, United Kingdom; ^25^ NIHR Biomedical Research Centre at Moorfields and UCL Institute of Ophthalmology, London, United Kingdom

**Keywords:** target product profile, TPP, quality by design, digital health technology, AI

## Abstract

Digital health technologies (DHTs), including those incorporating artificial intelligence (AI), have the potential to improve healthcare access, efficiency, and quality, reducing gaps between healthcare capacity and demand. Despite prioritisation in health policy, the adoption of DHTs remains limited, especially for AI, in part due to complex system requirements. Target product profiles (TPPs) are documents outlining the characteristics necessary for medical technologies to be utilised in practice and offer a way to align DHTs’ research and development with health systems’ needs. This systematic review examines current DHT TPPs’ methodologies, stakeholders, and contents. A total of 14 TPPs were identified, most targeted at low- and middle-income settings and communicable diseases. Only one TPP outlined the requirements for an AI device specifically. In total, 248 different characteristics were reported across the TPPs identified and were consolidated down to 33 key characteristics. Some considerations for DHTs’ successful adoption, such as regulatory requirements or environmental sustainability, were reported inconsistently or not at all. There was little standardisation in TPP development or contents, and limited transparency in reporting. Our findings emphasise the need for guidelines for TPP development, could help inform these, and could be used as a basis to develop future DHT TPPs.

**Systematic Review Registration**: https://www.researchprotocols.org/2024/1/e50568/authors.

## 1 Introduction

Digital health technologies (DHTs), including those incorporating artificial intelligence (AI), promise improved access, efficiency, and quality of healthcare, helping meet a growing mismatch between capacity and demand. Consequently, they have attracted significant public (1) and private investment (2), as well as prioritisation in health policy (3–5). The UK provides an example of a country with a strong political mandate to accelerate the adoption of DHTs and AI within the National Health Service (NHS) (1, 3), but where few have been integrated at scale (6). Many innovations fall into a widening implementation “gap” or “chasm” (7, 8) as they fail to meet the complex requirements of the wider UK health system (8–10). This is a problem shared with other countries (11, 12) but particularly pronounced in the UK, where multiple stakeholders are tasked with evaluating, implementing, and monitoring DHTs, including regulators, health technology assessment bodies, and local or national commissioners. These stakeholders' requirements can range from place-based evaluations of diagnostic or clinical utility (13), to cybersecurity (14) and environmental sustainability (15); however, many are poorly understood or defined, particularly for frontier technologies such as AI. This makes product development challenging, resulting in significant waste in research and development (16, 17).

Target product profiles (TPPs) offer a potential solution, providing a mechanism for health systems to “demand signal” to innovators. TPPs outline the desired characteristics of a product aimed at a particular disease or diseases (18). First utilised in the pharmaceutical industry, they have since been adapted by governments (19) and non-governmental organisations (NGOs) (18, 20) to outline the characteristics necessary for products to improve outcomes for patients and healthcare systems (21, 22) and enhance research efficiency. In a UK context, TPPs can fulfil key policy priorities to improve “demand signalling” (23), and facilitate wider digital transformation (3, 4) and innovation in life sciences (24). As a result, TPPs have attracted significant interest from key UK stakeholders, including the Medicines and Healthcare products Regulatory Agency (MHRA) (19), National Institute of Health and Care Excellence (NICE) (25), and Cancer Research UK (CRUK) (26, 27).

The absence of consensus on best practice for TPP development and contents presents a challenge to those seeking to develop them however (22, 28). Added to this, most TPPs to date have focused on *in vitro* diagnostics or therapeutics aimed at infectious diseases and low- and middle-income countries (LMICs) (22, 28), making their methods and contents potentially less generalisable to the UK context and DHTs, particularly those incorporating AI as or in a medical device.

This review aims to provide an overview of current DHT TPP methods, stakeholders, and contents to support the development of future such TPPs, including those that could be fulfilled by AI technologies.

## 2 Methods

This study is reported in line with the Preferred Reporting Items for Systematic review and Meta-Analyses (PRISMA) reporting guideline, with a checklist provided in Supplementary File S1. It was conducted in line with a protocol for our wider programme of work previously published (29).

MEDLINE, EMBASE, Web of Science (full collection and preprint), and ACM Digital Library were searched on 23 May 2023 using queries with search terms relevant to TPPs and DHTs such as “quality by design,” “target product profil*,” “QTPP*,” “digital health*,” “(online or web or internet or digital*),” and “(app or apps)” (see Supplementary File S2 for the full search strategy). Searches were developed using terms identified by an information specialist or published in previous systematic reviews (22) or online (30). No date or language limits were used.

A web search was conducted using methods outlined by Godin et al. (31) (see Supplementary File S1). Two researchers (TM and LV) performed Google searches and searched specific websites independently on 22 June 2023 and 8 July 2023, respectively, screening hits and their associated web pages. Records potentially relevant to the review were recorded on a spreadsheet on Microsoft Excel (Version 365; Microsoft Corporation, Redmond, WA, USA).

The online literature review platform Rayyan (Rayyan Systems, Cambridge, MA, USA) (32) was used to conduct this review. Rayyan's duplicate identification function was used to identify duplicates from the database search, which were then reviewed and removed manually from the list of potentially relevant records generated by manual web searches by one researcher (TBM). Two researchers (TBM and HDJH or LV) independently screened all remaining records by title and abstract and then full text against the inclusion and exclusion criteria. Records were included if they contained a TPP outlining minimum and/or desired characteristics for a product for use in healthcare and were for a DHT as defined by the NICE Evidence Standards Framework (ESF) for DHTs (13). References were excluded if they did not contain a TPP, the target product did not affect patient care (e.g., if it described a product or process used in pharmaceutical manufacture), or was not for a DHT as defined by the ESF. Disagreements between reviewers were resolved by discussion and arbitration by the senior author (AKD). The bibliographies of records included after the full-text screening were hand-searched for relevant references.

Two researchers (TM and HDJH) independently extracted information regarding the included TPPs and their development methods. The ESF was used to stratify target products into seven risk categories based on their potential risk to patients or healthcare systems: Tier A: System services; Tier B: Communicating about health and care; Tier B: Health and care diaries; Tier B: promoting good health; Tier C: Inform clinical management; Tier C: Drive clinical management; Tier C: Treat a specific condition; and Tier C: Diagnose a specific condition. The subdivision of Tier C aligns with the software as a medical device classification framework proposed by the International Medical Device Regulators Forum (33). The TPP development stages “scoping,” “drafting,” and “consensus-building” were taken from the study by Cocco et al. (22). Disagreements between reviewers were resolved by discussion and arbitration by the senior author (AD).

One researcher (TM) extracted all characteristics reported in previous TPPs, grouping these into the clusters “unmet clinical need,” “analytical performance,” “clinical validity,” “clinical utility,” “cost,” “environmental impact,” “regulatory requirements,” “human factors,” and “infrastructural requirements” outlined by Cocco et al. (22). Characteristics were deduplicated and consolidated by one researcher (TM), focusing on those relevant to software or in a medical device. All the characteristics originally reported, their clusters, consolidated characteristics, and exclusions were reviewed two other authors (HDJH and AKD). Disagreements were resolved by discussion and arbitration by the senior author (AKD).

Risk of bias assessments were not completed as no formal tools exist to assess TPPs.

## 3 Results

**Figure 1:**
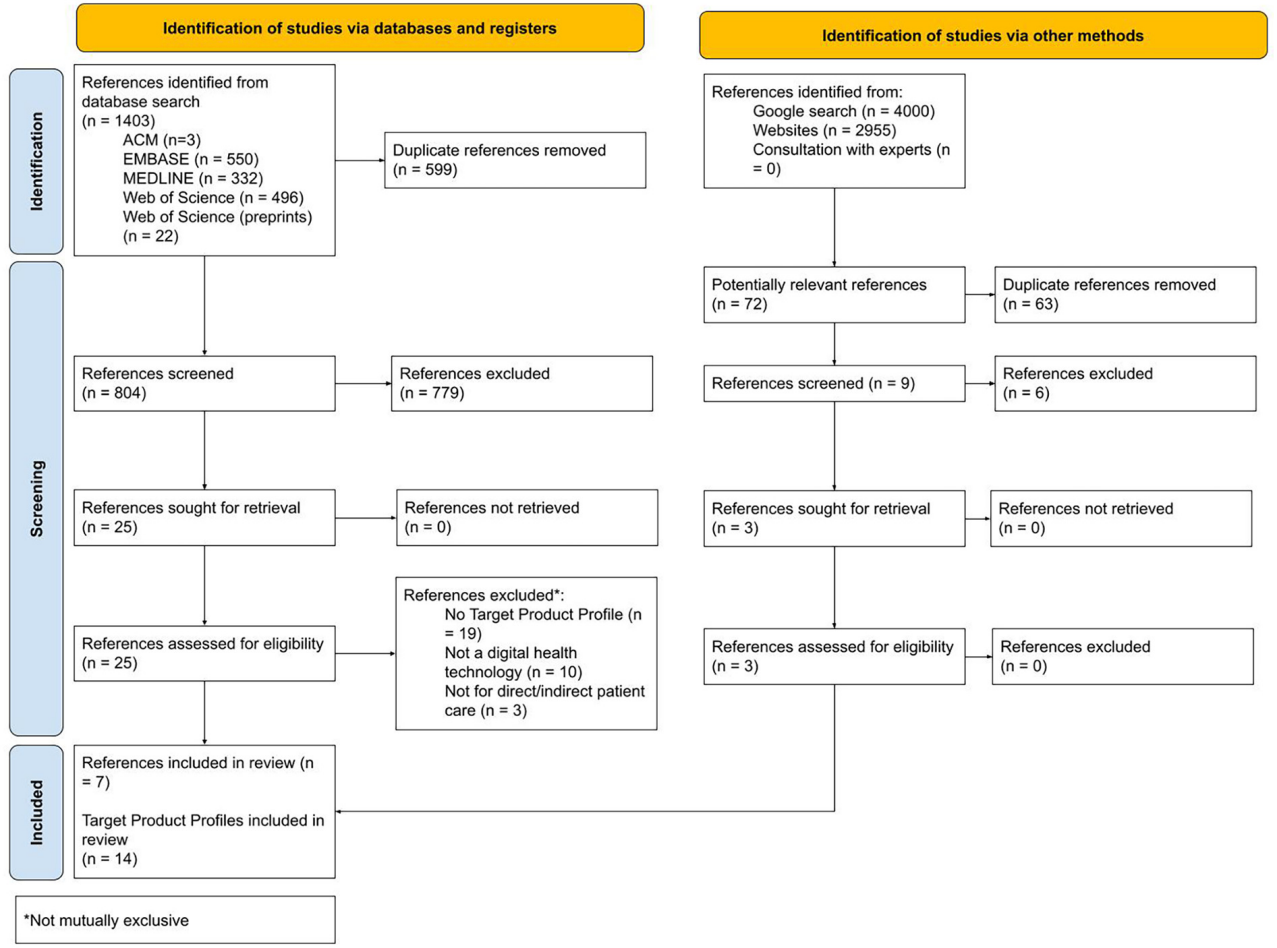
PRISMA flow diagram illustrating the results of the search and selection process.

Figure 1 outlines the results of the search and selection process.

### 3.1 Target product profile publication details and funding

Seven records met the inclusion criteria (34–40) and are listed in Table 1. Four records were identified from the database search (34, 36, 37, 39) and three from the Internet search (35, 38, 40). The publication year ranged from 2016 to 2023. Six records were journal articles (34, 36–40) and one was a PhD thesis (35). All were open access (34–40). A total of 14 TPPs were reported in the seven records. Falzon et al. included nine TPPs developed during the same study, eight of which met the inclusion criteria (34). Government agencies played a role in funding 12 (85.7%) TPPs. This was through USA (34) or UK (36, 37, 40) foreign aid, although one study (35) received funding from a UK research council. NGOs were involved in funding 10 (71.4%) TPPs (34, 37, 39), and universities funded 1 (7%). One TPP (7%) was funded solely by industry (38).

**Table 1:** Summary table of target product profile details.

Author	Title	Year	Disease area	Communicable or non-communicable target disease	LMIC or HIC target setting	Type of DHT	Funder(s)
Falzon et al.	Video treatment support for TB patients via mobiles	2016	TB	Communicable	Global health	Tier B: Communicating about health and care	NGO, government
Falzon et al.	eHealth portal to improve TB and tobacco care	2016	TB	Communicable	Global health	Tier A: System service	NGO, government
Falzon et al.	Digital dashboard for TB indicators and epidemiological trends	2016	TB	Communicable	Global health	Tier A: System service	NGO, government
Falzon et al.	Digital notification of TB cases	2016	TB	Communicable	Global health	Tier A: System service	NGO, government
Falzon et al.	Digital application for active TB drug safety monitoring	2016	TB	Communicable	Global health	Tier C: Inform clinical management	NGO, government
Falzon et al.	Diagnostic device connectivity for TB	2016	TB	Communicable	Global health	Tier A: System service	NGO, government
Falzon et al.	Information resources platform for patients on TB and smoking cessation	2016	TB	Communicable	Global health	Tier B: Promoting good health	NGO, government
Falzon et al.	Clinical decision support systems for TB treatment and smoking cessation	2016	TB	Communicable	Global health	Tier C: Drive clinical management	NGO, government
Keane et al.	Towards a Smartphone Connected Test for Influenza	2019	Influenza	Communicable	Global Health	Tier C: Diagnose a condition	Academia, government
Kadam et al.	Target Product Profile for a mobile app to read rapid diagnostic tests to strengthen infectious disease surveillance	2020	Infectious disease	Communicable	Global Health	Tier C: Drive clinical management	Government
Pelle et al.	Electronic clinical decision support algorithms incorporating point-of- care diagnostic tests in low-resource settings: a target product profile	2020	Primary care	Both	LMIC	Tier C: Drive clinical management	NGO, government
Ward et al.	The development of an artificial intelligence-based digital pathology for neglected tropical diseases: A platform specific analysis of the World Health Organization diagnostic target product profile for soil-transmitted helminthiasis	2022	Neglected tropical disease	Communicable	Not stated, LMICs inferred	Tier C: Diagnose a condition	Industry
White et al.	Target product profile for readers of rapid diagnostic tests	2023	Primary care	Not stated	Not stated, LMICs inferred	Tier C: Drive clinical management	NGOs
Tobin et al.	Development of a target product profile for a OneHealth antimicrobial resistance surveillance service	2023	Antimicrobial resistance	Communicable	Not stated, LMICs inferred	Tier A: System service	Government

Categories for type of DHT taken from NICE's Evidence Standards Framework for Digital Health Technologies. DHT, digital health technology; eHealth, electronic health; HIC, high-income country; LMIC, low- or middle-income country; NGO, non-governmental organisation; NICE, National Institute for Health and Care Excellence; TB, tuberculosis.

### 3.2 Target conditions, settings, and technologies

Of the 14 TPPs, 12 (85.7%) focused on infectious diseases (34, 35, 38, 40). Two targeted primary care, one to read rapid diagnostic tests (39) and the other provided clinical decision support (37), neither of which specified a communicable or non-communicable disease target. An LMIC target setting was explicit or implicit in all included TPPs, although some clinical problems could be seen as priorities for both high- and low-income countries, such as influenza (35) or antimicrobial resistance (40). Only one TPP specified the target product as being an AI device (38), although this was an adaptation of a technology agnostic TPP (41), rather than a *de novo* AI TPP.

Of the 14 TPPs, 5 (35.7%) outlined products with a NICE ESF Tier A risk classification (34, 40), 2 (14.3%) with a Tier B classification (34), and 7 (50%) with a Tier C classification (34–39). Of those in Tier C, one was classified as “inform clinical management” (34), four as “drive clinical management” (34, 36, 37, 39), and two as “diagnose a condition” (35, 38). No TPPs outlined a therapeutic target product.

### 3.3 Methods for target product profile development

Table 2 outlines included the TPPs’ development methods and participants. Scoping methods included expert opinion (40), “landscape analysis” (35), literature reviews (35, 36, 39), online questionnaires (34), and feedback gathered from pilots (40). The stakeholders involved in scoping were not clearly defined in 10/12 (71.4%) TPPs. In 4/14 (28.6%) TPPs where scoping stakeholders were defined, these comprised academics (35), NGOs (36, 37, 40), and clinicians (40). The number of participants involved in scoping was not stated in 12/14 (85.7%) TPPs, with two (36) and six (37) participants involved in scoping for the other two.

**Table 2:** Summary table of target product profile development methods and stakeholders.

Author	Scoping method	Scoping stakeholders	Scoping participant number	Drafting method	Drafting stakeholders	Drafting participant number	Consensus method	Consensus stakeholders	Consensus participant number
Falzon et al.	Online questionnaire	Not stated	Not stated	In-person meeting, online discussion	Not stated	Not stated	In-person meeting, online iteration (method unclear)	Not stated	Not stated
Falzon et al.	Online questionnaire	Not stated	Not stated	In-person meeting, online discussion	Not stated	Not stated	In-person meeting, online iteration (method unclear)	Not stated	Not stated
Falzon et al.	Online questionnaire	Not stated	Not stated	In-person meeting, online discussion	Not stated	Not stated	In-person meeting, online iteration (method unclear)	Not stated	Not stated
Falzon et al.	Online questionnaire	Not stated	Not stated	In-person meeting, online discussion	Not stated	Not stated	In-person meeting, online iteration (method unclear)	Not stated	Not stated
Falzon et al.	Online questionnaire	Not stated	Not stated	In-person meeting, online discussion	Not stated	Not stated	In-person meeting, online iteration (method unclear)	Not stated	Not stated
Falzon et al.	Online questionnaire	Not stated	Not stated	In-person meeting, online discussion	Not stated	Not stated	In-person meeting, online iteration (method unclear)	Not stated	Not stated
Falzon et al.	Online questionnaire	Not stated	Not stated	In-person meeting, online discussion	Not stated	Not stated	In-person meeting, online iteration (method unclear)	Not stated	Not stated
Falzon et al.	Online questionnaire	Not stated	Not stated	In-person meeting, online discussion	Not stated	Not stated	In-person meeting, online iteration (method unclear)	Not stated	Not stated
Keane et al.	Landscape analysis, literature reviews, prior TPPs	Academia	Not stated	Not stated	Academia, NGO	10	Not performed	n/a	n/a
Kadam et al.	Literature review	NGO	2	Not stated	NGO	2	Delphi-like process	NGOs, industry, academia, consultants	51
Pelle et al.	Adaptation of standard TPP frameworks (FIND and WHO)	NGOs, government	6	Meeting	Academia, industry, government	39	Delphi-like process	NGOs, academia, government, clinicians	28 first round, 23 second round
Ward et al.	Adapted a technology agnostic TPP	Not stated	Not stated	Not stated	NGO, industry, academic, government	13	Not performed	Not performed	Not performed
White et al.	WHO TPP framework, literature review	Not stated	Not stated	Not stated	Not stated	Not stated	e-Delphi in expert group, public consultation, final review by expert group	NGO, clinicians, government, industry	40 participated in e-Delphi, 27 responded to public consultation, 28 participated in final review by expert group
Tobin et al.	WHO TPP framework, pilot development, engagement with development community	Clinicians, NGOs, developers	Not stated	Not stated	Not stated	Not stated	e-Delphi	Industry, NGOs, academia, government, clinicians	43 first round, second round not stated

FIND, foundation for innovative new diagnostics; NGO, non-governmental organisation; TB, tuberculosis; TPP, target product profile; WHO, World Health Organization.

Of the 14 TPPs, characteristics for 6 (42.8%) were drafted through a meeting (34, 37) with the drafting method not stated in 8 (57.1%). Stakeholders involved in drafting included academics (35, 37, 38), government (37, 38), industry (38), and NGOs (35, 36, 38); however, 10/14 (71.4%) TPPs did not state the stakeholders involved. The number of stakeholders involved in drafting was stated in 4/14 (28.5%) TPPs (35–38) and ranged from two (36) to 39 (37) participants.

The use of a consensus method was reported in 12/14 (85.6%) TPPs (34, 36, 37, 39, 40). Four TPPs (28.5%) stated using either a “Delphi-like process” (36, 37) or “e-Delphi” (39, 40). Maximum Delphi round participant numbers were 28 (37), 40 (39), 43 (40), and 51 (36). Participants included academics (36, 37, 40), clinicians (37, 40), consultants (36), industry (36, 39, 40), government (37, 39, 40), and NGOs (36, 37, 39, 40). The consensus method was an in-person meeting and online iteration (methodology unclear) in 8/14 (57.1%) TPPs (34), although the number of participants and their stakeholder groups were not stated.

### 3.4 Characteristics reported in target product profiles

The TPPs reported 248 different characteristics (see Supplementary Table S1). Of the 14 TPPs, all reported characteristics in the clusters “unmet clinical need” and “infrastructural requirements,” 13 (92.9%) reported characteristics in the “human factors,” 11 (78.6%) in the clusters “clinical utility,” “costs,” and “regulatory requirements,” 8 (57.1%) in “clinical validity,” and 5 (35.7%) in “analytic performance” (see Table 3). None reported characteristics in “environmental impact.”

**Table 3:** Clusters and consolidated characteristics reported in previous target product profiles (TPPs) for digital health technologies.

Cluster	TPPs reporting cluster [*n* (%)]	Consolidated characteristics	TPPs reporting consolidated characteristic [*n* (%)]	Characteristics reported in TPPs forming consolidated characteristic (*n*)	Frequency constituent characteristics reported^a^ (*n*)
Unmet clinical need	14 (100)	Aggregate data reporting	3 (21)	7	7
		Input data	7 (50)	27	29
		Operating modes	1 (7)	1	1
		Output data	5 (36)	11	11
		Pathway position	3 (21)	3	11
		Purpose	14 (100)	3	14
		Target end-user	13 (93)	6	13
		Target population	4 (29)	2	4
		Target setting	5 (36)	9	12
Clinical validity	8 (57)	Diagnostic performance	8 (57)	13	15
Clinical utility	11 (79)	Effect on clinical outcomes	8 (57)	5	12
		Effect on service outcomes	4 (29)	5	5
		Features to facilitate research	1 (7)	1	1
Analytic performance	5 (36)	Scalability	1 (7)	1	1
Costs	11 (79)	Cost	11 (79)	10	13
		Intellectual property	3 (21)	1	3
		Product lead time	1 (7)	1	1
Environmental impact	0 (0)				
Regulatory requirements	11 (79)	Data governance and security	9 (64)	22	33
		Monitoring	2 (14)	2	2
		Regulatory requirements	6 (43)	6	8
		System malfunction protection	1 (7)	1	1
Human factors	13 (93)	Acceptability with stakeholders	3 (21)	3	3
		Customisability	6 (43)	5	7
		Data dictionary	2 (14)	1	2
		Interface	11 (79)	20	26
		Language	4 (29)	2	4
		Product support	6 (43)	7	7
		Social factors	1 (7)	1	1
		Training	7 (50)	8	11
Infrastructural requirements	14 (100)	Compatibility with software	11 (79)	15	23
		Compatibility with hardware	5 (36)	16	16
		Connectivity	8 (57)	8	9
		Interoperability	1 (7)	1	1

All the characteristics reported in previous TPPs were extracted (see Supplementary Table S1), deduplicated, and consolidated to produce the “consolidated characteristics.” Characteristics reported in previous digital health technology TPPs were excluded if they were not relevant to software as or in a medical device. “Constituent characteristic” refers to the original characteristics reported in by the TPPs.

^a^ The frequency for constituent characteristics being reported is at times greater than the number of TPPs included in the review due to the combination of multiple constituent characteristics into “consolidated characteristics.”

The number of characteristics reported in previous TPPs was reduced to 33 after deduplication, consolidation, and exclusion (see Table 3). Supplementary Table S1 outlines the destination of each originally reported characteristic.

## 4 Discussion

To our knowledge, this study represents the first systematic review of TPPs for DHTs and is of particular relevance given the increasing interest in these technologies’ wider adoption. We adapted established methods to create a robust strategy for the identification and evaluation of TPPs. Those included predominantly focus on LMICs and lack transparency and patient and end-user input in their development. Standardisation of TPP methods, contents, and transparency is strikingly lacking. Despite this, the identified TPPs consistently report a range of characteristics that could form the basis of future TPP development.

We used established, peer-reviewed methods for the identification, categorisation, and evaluation of TPPs for DHTs, increasing our review's comprehensiveness, robustness, and reliability. The terms used to identify records in bibliographic databases were developed in consultation with an information specialist and included combinations of terms to identify TPPs for DHTs as varied as health informatics solutions, electronic health records, software as a medical device, apps, artificial intelligence, and telemedicine. The filters used to identify TPPs have been published previously in peer-reviewed literature (22), while our Internet search strategy used established methods (31) and resulted in the identification of a further three TPPs. NICE's ESF was used to categorise target technologies and was developed through an extensive consensus process (13), while the methods used to categorise TPPs’ development and characteristics have been published previously after peer review (22). TPPs were evaluated by two researchers working independently. This approach is likely to have identified the majority of TPPs for DHTs published up to the search dates, with their methods and contents evaluated in a robust, reliable, and unbiased way.

Every TPP identified by this review focused on LMICs and predominantly communicable diseases. These are findings similar to previous reviews for diagnostic tests (22) and medical technologies in general (including therapeutics) (28). Although unsurprising given TPPs’ prior utilisation and championing by NGOs with a LMIC/global health focus (18, 20, 42), this potentially makes their contents and characteristics less generalisable to high-income country (HIC) contexts.

We found a lack of clear, transparent reporting of TPPs’ development methods and participants, again echoing the findings of previous reviews (22, 28). Recognising TPPs’ noble ambitions to draw funding towards neglected diseases and contexts, and that TPP research is likely to be similarly under-resourced, this lack of transparency makes critical appraisal challenging and undermines DHT TPPs’ reliability and comprehensiveness. Greater transparency is particularly important if TPPs are to be used in HICs, as this would represent a significant opportunity for regulatory or policy capture (43). We therefore echo previous calls for standardisation in TPP methods, contents, and reporting (22, 28) to improve transparency. Although a World Health Organization (WHO) TPP generation process was utilised by a number of included TPPs (37–40), this document is not in the public domain. TPPs developed using this process used Delphi methods, an established and validated consensus process; however, TPP development would benefit from formal guidelines for development and reporting published open access, similar to those published for reporting guidelines (44) and core outcome sets (45).

A key output of this review is a list of characteristics reported in previous DHT TPPs (see Table 3; Supplementary Table S1). Although the number of TPPs we identified was relatively small (*n* = 14), they reported 248 different characteristics. Their consolidation down to 33 characteristics (Table 3) suggests a reassuring level of consistency in TPPs’ scope and contents, although the reporting of considerations key to the successful use of DHTs was inconsistent. This included key elements, such as target population (4/14, 28.6%) and pathway position (3/14, 21.4%) [key components of an intended use statement (46)], to effects on clinical and service outcomes (8/14, 57.1% and 4/14, 28.6%, respectively), data governance and security (9/14, 64.3%), and regulatory requirements (6/14, 42.9%). Guidelines for DHT TPPs’ development and contents could help address these gaps in future, improving such documents’ comprehensiveness and reliability.

Despite variability in the scope and comprehensiveness of individual TPPs, the input of stakeholders with significant knowledge and expertise in DHT development and implementation to TPP development, such as government agencies (34–37, 40), NGOs (34, 37, 39), and industry (38), means the characteristics they report as a whole are likely to be fairly comprehensive. Using these as the basis for future TPPs’ development may therefore ensure future TPPs have sufficient scope and granularity.

Before doing so, however, it is important to consider if key characteristics may have been omitted from previous DHT TPPs as a whole. Using the UK context as an example, comparison to relevant policy documents, such as those used to guide health technology assessments (13) or AI procurement (47), highlights significant gaps. This includes factors such as DHTs’ environmental sustainability, with digital transformation set to play a key role in fulfilling the NHS’ commitment to net zero by 2045 (48); social value, an essential part of government procurement and commissioning (15); and effects on health inequalities, a persistent UK policy concern and priority (4). These are concerns and considerations shared with other HICs (49–51). TPPs for DHTs that may be met by AI technologies should also address concerns regarding AI's potential for algorithmic bias (52) and performance changes over time (53), taking into account requirements utilised to mitigate these in evaluation and implementation (54–56).

TPPs must reflect the needs of end users to be of utility. Only 3/14 (21.4%) TPPs stated that clinicians were involved in their development (37, 39, 40) and none stated that they involved patients in the development process. Although this would have been challenging given many TPPs’ supranational focus, end-user and patient involvement is essential for the development of future documents, particularly in HIC contexts. In the UK, patient involvement is essential in healthcare research and priority setting (13, 47, 57), a position increasingly adopted in other HICs (58–60). Patient involvement in DHT TPP development is essential not only because DHTs may affect patient care or handle sensitive information, but because these technologies must meet wider public expectations to be sustainably adopted (6). This is particularly relevant as many DHTs are seen as a means to empower patients to better manage their own health (61), meaning patients may be the target product's end user.

As well as significant patient, public, and end-user involvement, the development of future TPPs for specific national contexts would likely benefit from the close involvement of relevant regulators, health technology assessment bodies, and healthcare systems. These stakeholders could seek to develop their own TPPs, a role similar to that taken by WHO and other NGOs in LMIC settings or by the UK's MHRA during the COVID-19 pandemic, when it signalled to industry the UK's demand for such tests as well as the agency's likely product requirements. Alternatively, these stakeholders may wish to contribute to the TPP development processes led by others, such as academia or patient advocacy bodies. This could be by providing legislative requirements, standards, or guidance in general, or recommendations tailored to a specific product or disease area. Given regulators’, health technology assessment bodies’, and healthcare systems’ crucial role in approving, commissioning, and monitoring DHTs, their involvement is likely to be crucial to impart these documents with sufficient accuracy and authority, particularly in HIC settings.

### 4.1 Limitations

This study has several limitations. First, publicly available TPPs likely represent a fraction of those developed, with many internal to pharmaceutical or medical device companies (21, 28) and therefore not in the public domain. These documents may offer more refined methods, characteristics, or best practice, not captured by this review.

In addition, much of our assessment of TPPs’ details, methods, and characteristics was subjective. Although two researchers performed data extraction and analysis to mitigate this, there remains a residual risk of misinterpretation and bias, particularly as TPPs were often poorly reported. Scoping, drafting, and consensus methods and participants were often hard to identify, with information having to be pieced together from limited information in the manuscript. For example, stakeholder involvement often had to be deciphered from authors’ affiliations. It is possible that a doctor specialising in infectious diseases could be a clinician, academic, member of governmental or non-governmental organisations, or industry consultant, with an affiliation provided to only one or a limited number of these, thus making our judgement of stakeholder involvement less accurate. This further strengthens the argument for increased standardisation and transparency in TPPs’ development and reporting.

Finally, the inclusion of TPPs for DHTs integrated within or working downstream of *in-vitro* diagnostics (IVDs), such as lateral flow tests, introduces the potential for misinterpretations of characteristics. For example, terms like “sensitivity” and “calibration” may differ in meaning between DHT and IVD contexts. “Sensitivity” may refer to analytical or diagnostic sensitivity, while “calibration” may refer to setting or adjusting the measurement system of a laboratory instrument or assay, or the agreement between the predicted and actual observed outcomes for a prognostic DHT. Confusion could be avoided in future with agreed definitions for these terms included in DHT TPP development guidelines.

## 5 Conclusions

This review highlights the current state of the art in the development and contents of DHT TPPs, as recorded in the medical and grey literature. It has found significant weaknesses in TPPs’ methods, contents, and reporting, emphasising the need for greater standardisation and transparency. This review could inform best practice or formal reporting guidelines for TPPs. In addition, we report a list of characteristics distilled from existing DHT TPPs that could provide a starting point for the development of similar documents in future, including those incorporating AI.

## Data Availability

The original contributions presented in the study are included in the article/Supplementary Material, further inquiries can be directed to the corresponding author.
