# Supplementary material for: Target product profiles for digital health technologies including those with artificial intelligence: a systematic review
Source: Front Health Serv. 2025 May 20;5:1537016. doi: 10.3389/frhs.2025.1537016 (PMC12131871; doi:10.3389/frhs.2025.1537016)
Supplement: Supplementary file 3 [file Datasheet1.docx]

# Search strategy

## MEDLINE

Ovid MEDLINE(R) ALL

1 "quality by design".ti,ab,kw.

2 "target product profil*".ti,ab,kw.

3 QTPP*.ab,kw,ti.

4 or/1-3

5 exp Internet/

6 (online or web or internet or digital*).ti,ab,kw.

7 ((online or web or internet or digital*) adj3 (based or application* or intervention* or program* or therap*)).ti,ab,kw.

8 exp Informatics/

9 exp Medical Informatics/

10 exp Nursing informatics/

11 exp Medical Informatics Computing/

12 exp Consumer Health Informatics/

13 exp Public Health Informatics/

14 exp Medical Informatics Applications/

15 informatics.ti,ab,kw.

16 exp Information Technology/

17 exp Electronic Health Records/

18 exp Medical Records Systems, Computerized/

19 Automated Medical Record*.ti,ab,kw.

20 computeri#ed medical record*.ti,ab,kw.

21 EHR*.ti,ab,kw.

22 Electronic Medical Record*.ti,ab,kw.

23 electronic patient record*.ti,ab,kw.

24 personal health record*.ti,ab,kw.

25 electronic health record*.ti,ab,kw.

26 EMR*.ti,ab,kw.

27 personal health record*.ti,ab,kw.

28 exp Health Information Systems/

29 health information system*.ti,ab,kw.

30 exp Information Systems/

31 exp Computers/

32 exp Precision Medicine/

33 precision medicine.ti,ab,kw.

34 exp Software/

35 software.ti,ab,kw.

36 SaMD.ti,ab,kw.

37 exp Big Data/

38 big data.ti,ab,kw.

39 exp Therapy, Computer-Assisted/

40 exp internet-based intervention/ or social media/

41 exp Digital Technology/

42 virtual health*.ti,ab,kw.

43 health technolog*.ti,ab,kw.

44 "health 2.0".ti,ab,kw.

45 (cyber-medicine* or cybermedicine*).ti,ab,kw.

46 (epatient* or e-patient*).ti,ab,kw.

47 digital health*.ti,ab,kw.

48 (personal adj3 digital).ti,ab,kw.

49 digital medicine*.ti,ab,kw.

50 or/5-49

51 exp Mobile Applications/

52 (app or apps).ti,ab,kw.

53 (mobile application* or App-based).ti,ab,kw.

54 51 or 52 or 53

55 exp Artificial Intelligence/

56 ((artificial* or deep*) adj1 (intelligen* or learn* or smart*)).ti,ab,kw.

57 ((computat* or computer* or machine) adj1 (intelligen* or smart*)).ti,ab,kw.

58 (expert system* adj3 (intelligen* or smart*)).ti,ab,kw.

59 ((Artificial* or intelligen*) adj2 (comput* or technol* or network* or imaging or digital*)).ti,ab,kw.

60 Generative adversarial network.ti,ab,kw.

61 (Algorith* adj5 (intelligen* or artificial* or smart* or automat*)).ti,ab,kw.

62 (Intelligen* adj2 (analys* or analyz* or system* or screen* or informatic* or application* or app or apps or process* or simulation* or interactive* or interaction*)).ti,ab,kw.

63 ((decision tree* or random forest* or knowledge representation* or computer vision system* or computer reasoning* or natural language processing* or connectionist model*) adj2 artificial*).ti,ab,kw.

64 (AI not (aromatase inhibitor* or anatomic insertion* or AI-IgG or apnea index or anti-infective or angiogenesis inhibitors)).ti,ab,kw.

65 (machinelearn* or machine-learn*).ti,ab,kw.

66 exp neural networks, computer/

67 (neural network* adj1 (artificial* or convolutional or deep*)).ti,ab,kw.

68 exp Diagnosis, Computer-Assisted/ and (intelligen* or smart or artificial*).ti,ab,kw.

69 exp Diagnosis, Computer-Assisted/ and (accura* adj2 (diagnos* or detect* or assess* or class*)).ti,ab,kw.

70 exp Diagnosis, Computer-Assisted/ and (automat* adj2 (system* or CAD system* or methodolog* or screen* or identif* or local* or class* or detect* or diagnos*)).ti,ab,kw.

71 exp Therapy, Computer-Assisted/ and (intelligen* or smart or artificial* or automat*).ti,ab,kw.

72 ((assist* or aid* or diagnos* or screen*) adj2 (intelligen* or artificial* or smart* or automat*)).ti,ab,kw.

73 55 or 56 or 57 or 58 or 59 or 60 or 61 or 62 or 63 or 64 or 65 or 66 or 67 or 68 or 69 or 70 or 71 or 72

74 exp Telemedicine/

75 (telemedicine* or tele-medicine* or remote medicine*).ti,ab,kw.

76 (tele-health* or telehealth* or remote health*).ti,ab,kw.

77 (telecare* or tele-care* or remote care).ti,ab,kw.

78 exp Remote Consultation/

79 ((remote* or tele*) adj2 (patient monitor* or consult* or deliver* or intervention* or treatment* or diagnos* or service* or program* or prevent*)).ti,ab,kw.

80 exp Videoconferencing/

81 (videoconferenc* or video-conferenc* or videoconsult* or video-consult*).ti,ab,kw.

82 or/74-81

83 exp Cell Phone/

84 (phone* or telephone* or smartphone* or cellphone* or cell-phone* or smartwatch*).ti.

85 ((phone* or telephone* or smartphone* or cellphone* or smartwatch*) adj3 (based or application* or intervention* or program* or therap*)).ab.

86 (Smartphone* or smart-phone*).ti,ab,kw.

87 (Cell-phone* or cellphone*).ti,ab,kw.

88 Mobile-phone*.ti,ab,kw.

89 exp Computers, Handheld/

90 exp Text Messaging/

91 ((text* or txt) adj3 message*).ti,ab,kw.

92 (SMS or MMS).ti,ab,kw.

93 mhealth.ti,ab,kw.

94 mobile comput*.ti,ab,kw.

95 (m-health or mhealth or m-therapy or mtherapy or mobile health*).ti,ab,kw.

96 ((mobile* or internet*) adj2 intervention*).ti,ab,kw.

97 (mobile health or mhealth or m-health or ehealth or e-health or emental or e-mental).ti.

98 ((mobile health or mhealth or m-health or ehealth or e-health or emental or e-mental) adj3 (based or application* or intervention* or program* or therap*)).ab.

99 (mobile* adj3 (based or application* or intervention* or device* or technolog*)).ti,ab,kw.

100 ((mhealth or m-health or ehealth or e-health or technology enabled care or digital health* or digital medicine* or eportal* or e-portal* or digital portal* or virtual health* or mobile health* or mobile intervention* or mobile comput* or Internet of Things or IoT or epatient* or e-patient* or online or web* or internet or zoom) adj3 (service* or program* or intervention* or deliver* or remote* or treatment* or diagnos* or prevent* or therap* or setting* or session* or remote* or train* or technolog*)).ti,ab,kw.

101 mobile health*.ti,ab,kw.

102 mobile intervention*.ti,ab,kw.

103 (mobile adj3 (comput*or device* or application* or technolog* or platform* or software*)).ti,ab,kw.

104 ((App or Apps) not (mutant or mice or amyloid precursor protein* or gene dose* or acute phase protein*)).ti,ab,kw.

105 (tablet* adj3 (application* or device* or technolog* or platform* or mobile* or comput* or software*)).ti,ab,kw.

106 (mobile application* or Appstore or App-based).ti,ab,kw.

107 or/83-106

108 exp Wearable Electronic Devices/

109 wearable*.ti,ab,kw.

110 108 or 109

111 50 or 54 or 73 or 82 or 107 or 110

112 4 and 111

## EMBASE

1. "quality by design".ti,ab,kw.

2. "target product profil*".ti,ab,kw.

3. QTPP*.ab,kw,ti.

4. or/1-3

5. exp Internet/

6. (online or web or internet or digital*).ti,ab,kw.

7. ((online or web or internet or digital*) adj3 (based or application* or intervention* or program* or therap*)).ti,ab,kw.

8. exp information science/

9. exp medical informatics/

10. exp nursing informatics/

11. exp consumer health informatics/

12. informatics.ti,ab,kw.

13. exp information technology/

14. exp electronic health record/

15. electronic medical record system/

16. exp medical information system/

17. health information system*.ti,ab,kw.

18. exp computer/

19. exp personalized medicine/

20. exp software/

21. software.ti,ab,kw.

22. SaMD.ti,ab,kw.

23. exp big data/

24. big data.ti,ab,kw.

25. exp computer assisted therapy/

26. exp internet-based intervention/ or social media/

27. exp Digital Technology/

28. virtual health*.ti,ab,kw.

29. health technolog*.ti,ab,kw.

30. "health 2.0".ti,ab,kw.

31. (cyber-medicine* or cybermedicine*).ti,ab,kw.

32. (epatient* or e-patient*).ti,ab,kw.

33. digital health*.ti,ab,kw.

34. (personal adj3 digital).ti,ab,kw.

35. digital medicine*.ti,ab,kw.

36. Automated Medical Record*.ti,ab,kw.

37. computeri#ed medical record*.ti,ab,kw.

38. EHR*.ti,ab,kw.

39. Electronic Medical Record*.ti,ab,kw.

40. electronic patient record*.ti,ab,kw.

41. personal health record*.ti,ab,kw.

42. electronic health record*.ti,ab,kw.

43. EMR*.ti,ab,kw.

44. personal health record*.ti,ab,kw.

45. or/5-44

46. exp mobile application/

47. (app or apps).ti,ab,kw.

48. (mobile application* or App-based).ti,ab,kw.

49. 46 or 47 or 48

50. exp artificial intelligence/

51. ((artificial* or deep*) adj1 (intelligen* or learn* or smart*)).ti,ab,kw.

52. ((computat* or computer* or machine) adj1 (intelligen* or smart*)).ti,ab,kw.

53. (expert system* adj3 (intelligen* or smart*)).ti,ab,kw.

54. ((Artificial* or intelligen*) adj2 (comput* or technol* or network* or imaging or digital*)).ti,ab,kw.

55. Generative adversarial network.ti,ab,kw.

56. (Algorith* adj5 (intelligen* or artificial* or smart* or automat*)).ti,ab,kw.

57. (Intelligen* adj2 (analys* or analyz* or system* or screen* or informatic* or application* or app or apps or process* or simulation* or interactive* or interaction*)).ti,ab,kw.

58. ((decision tree* or random forest* or knowledge representation* or computer vision system* or computer reasoning* or natural language processing* or connectionist model*) adj2 artificial*).ti,ab,kw.

59. (AI not (aromatase inhibitor* or anatomic insertion* or AI-IgG or apnea index or anti-infective or angiogenesis inhibitors)).ti,ab,kw.

60. (machinelearn* or machine-learn*).ti,ab,kw.

61. exp artificial neural network/

62. (neural network* adj1 (artificial* or convolutional or deep*)).ti,ab,kw.

63. exp Diagnosis, Computer-Assisted/ and (intelligen* or smart or artificial*).ti,ab,kw.

64. exp Diagnosis, Computer-Assisted/ and (intelligen* or smart or artificial*).ti,ab,kw.

65. exp Diagnosis, Computer-Assisted/ and (automat* adj2 (system* or CAD system* or methodolog* or screen* or identif* or local* or class* or detect* or diagnos*)).ti,ab,kw.

66. exp Therapy, Computer-Assisted/ and (intelligen* or smart or artificial* or automat*).ti,ab,kw.

67. ((assist* or aid* or diagnos* or screen*) adj2 (intelligen* or artificial* or smart* or automat*)).ti,ab,kw.

68. or/50-67

69. exp telemedicine/

70. (telemedicine* or tele-medicine* or remote medicine*).ti,ab,kw.

71. (tele-health* or telehealth* or remote health*).ti,ab,kw.

72. (tele-health* or telehealth* or remote health*).ti,ab,kw.

73. exp teleconsultation/

74. ((remote* or tele*) adj2 (patient monitor* or consult* or deliver* or intervention* or treatment* or diagnos* or service* or program* or prevent*)).ti,ab,kw.

75. exp videoconferencing/

76. (videoconferenc* or video-conferenc* or videoconsult* or video-consult*).ti,ab,kw.

77. 69 or 70 or 71 or 72 or 73 or 74 or 75 or 76

78. exp mobile phone/

79. (phone* or telephone* or smartphone* or cellphone* or cell-phone* or smartwatch*).ti,ab,kw.

80. ((phone* or telephone* or smartphone* or cellphone* or smartwatch*) adj3 (based or application* or intervention* or program* or therap*)).ti,ab,kw.

81. (Smartphone* or smart-phone*).ti,ab,kw.

82. (Cell-phone* or cellphone*).ti,ab,kw.

83. Mobile-phone*.ti,ab,kw.

84. exp personal digital assistant/

85. exp text messaging/

86. ((text* or txt) adj3 message*).ti,ab,kw.

87. (SMS or MMS).ti,ab,kw.

88. mhealth.ti,ab,kw.

89. mobile comput*.ti,ab,kw.

90. mobile comput*.ti,ab,kw.

91. (m-health or mhealth or m-therapy or mtherapy or mobile health*).ti,ab,kw.

92. ((mobile* or internet*) adj2 intervention*).ti,ab,kw.

93. ((mobile* or internet*) adj2 intervention*).ti,ab,kw.

94. ((mobile health or mhealth or m-health or ehealth or e-health or emental or e-mental) adj3 (based or application* or intervention* or program* or therap*)).ti,ab,kw.

95. (mobile* adj3 (based or application* or intervention* or device* or technolog*)).ti,ab,kw.

96. ((mhealth or m-health or ehealth or e-health or technology enabled care or digital health* or digital medicine* or eportal* or e-portal* or digital portal* or virtual health* or mobile health* or mobile intervention* or mobile comput* or Internet of Things or IoT or epatient* or e-patient* or online or web* or internet or zoom) adj3 (service* or program* or intervention* or deliver* or remote* or treatment* or diagnos* or prevent* or therap* or setting* or session* or remote* or train* or technolog*)).ti,ab,kw.

97. mobile health*.ti,ab,kw.

98. mobile intervention*.ti,ab,kw.

99. (mobile adj3 (comput*or device* or application* or technolog* or platform* or software*)).ti,ab,kw.

100. ((App or Apps) not (mutant or mice or amyloid precursor protein* or gene dose* or acute phase protein*)).ti,ab,kw.

101. (tablet* adj3 (application* or device* or technolog* or platform* or mobile* or comput* or software*)).ti,ab,kw.

102. (mobile application* or Appstore or App-based).ti,ab,kw.

103. 78 or 79 or 80 or 81 or 82 or 83 or 84 or 85 or 86 or 87 or 88 or 89 or 90 or 91 or 92 or 93 or 94 or 95 or 96 or 97 or 98 or 99 or 100 or 101 or 102

104. exp wearable computer/

105. wearable*.ti,ab,kw.

106. 104 or 105

107. 45 or 49 or 68 or 77 or 103 or 106

108. 4 and 107

## Web of Science (core collection)

# Entitlements:

- WOS.IC: 1993 to 2023

- WOS.CCR: 1985 to 2023

- WOS.SCI: 1900 to 2023

- WOS.AHCI: 1975 to 2023

- WOS.BHCI: 2010 to 2023

- WOS.BSCI: 2010 to 2023

- WOS.ESCI: 2015 to 2023

- WOS.ISTP: 1990 to 2023

- WOS.SSCI: 1900 to 2023

- WOS.ISSHP: 1990 to 2023

# Searches:

1: (TI =("quality by design")) OR (AB =("quality by design")) OR (AK =("quality by design"))

2: (TI =("target product profil*")) OR (AB =("target product profil*")) OR (AK =("target product profil*")

3: (TI =QTPP*) OR (AB =QTPP*) OR (AK =QTPP*)

4: #3 OR #2 OR #1

5: (TI =(online or web or internet or digital*)) OR (AB =(online or web or internet or digital*)) OR (AK =(online or web or internet or digital*))

6: (TI =((online or web or internet or digital*) NEAR/3 (based or application* or intervention* or program* or therap*))) OR (AB =((online or web or internet or digital*) NEAR/3 (based or application* or intervention* or program* or therap*))) OR (AK =((online or web or internet or digital*) NEAR/3 (based or application* or intervention* or program* or therap*)))

7: (TI =informatics) OR (AB =informatics) OR (AK =informatics)

8: (TI =Automated Medical Record*) OR (AB =Automated Medical Record*) OR (AK =Automated Medical Record*)

9: (TI =computeri$ed medical record*) OR (AB =computeri$ed medical record*) OR (AK =computeri$ed medical record*)

10: (TI =EHR*) OR (AB =EHR*) OR (AK =EHR*)

11: (TI =Electronic Medical Record*) OR (AB =Electronic Medical Record*) OR (AK =Electronic Medical Record*)

12: (TI =electronic patient record*) OR (AB =electronic patient record*) OR (AK =electronic patient record*)

13: (TI =personal health record*) OR (AB =personal health record*) OR (AK =personal health record*)

14: (TI =electronic health record*) OR (AB =electronic health record*) OR (AK =electronic health record*)

15: (TI =EMR*) OR (AB =EMR*) OR (AK =EMR*)

16: (TI =personal health record*) OR (AB =personal health record*) OR (AK =personal health record*)

17: (TI =health information system*) OR (AB =health information system*) OR (AK =health information system*)

18: (TI =precision medicine) OR (AB =precision medicine) OR (AK =precision medicine)

19: (TI =software) OR (AB =software) OR (AK =software)

20: (TI =SaMD) OR (AB =SaMD) OR (AK =SaMD)

21: (TI =big data) OR (AB =big data) OR (AK =big data)

22: (TI =virtual health*) OR (AB =virtual health*) OR (AK =virtual health*)

23: (TI =health technolog*) OR (AB =health technolog*) OR (AK =health technolog*)

24: (TI ="health 2.0") OR (AB ="health 2.0") OR (AK ="health 2.0")

25: (TI =(cyber-medicine* or cybermedicine*)) OR (AB =(cyber-medicine* or cybermedicine*)) OR (AK =(cyber-medicine* or cybermedicine*))

26: (TI =(epatient* or e-patient*)) OR (AB =(epatient* or e-patient*)) OR (AK =(epatient* or e-patient*))

27: (TI =digital health*) OR (AB =digital health*) OR (AK =digital health*)

28: (TI =(personal NEAR/3 digital)) OR (AB =(personal NEAR/3 digital)) OR (AK =(personal NEAR/3 digital))

29: (TI =digital medicine*) OR (AB =digital medicine*) OR (AK =digital medicine*)

30: #5 OR #6 OR #7 OR #8 OR #9 OR #10 OR #11 OR #12 OR #13 OR #14 OR #15 OR #16 OR #17 OR #18 OR #19 OR #20 OR #21 OR #22 OR #23 OR #24 OR #25 OR #26 OR #27 OR #28 OR #29

31: (TI =(app or apps)) OR (AB =(app or apps)) OR (AK =(app or apps))

32: (TI =(mobile application* or App-based)) OR (AB =(mobile application* or App-based)) OR (AK =(mobile application* or App-based))

33: #32 OR #31

34: (TI =((artificial* or deep*) NEAR/1 (intelligen* or learn* or smart*))) OR (AB =((artificial* or deep*) NEAR/1 (intelligen* or learn* or smart*))) OR (AK =((artificial* or deep*) NEAR/1 (intelligen* or learn* or smart*)))

35: (TI =((computat* or computer* or machine) NEAR/1 (intelligen* or smart*))) OR (AB =((computat* or computer* or machine) NEAR/1 (intelligen* or smart*))) OR (AK =((computat* or computer* or machine) NEAR/1 (intelligen* or smart*)))

36: (TI =(expert system* NEAR/3 (intelligen* or smart*))) OR (AB =(expert system* NEAR/3 (intelligen* or smart*))) OR (AK =(expert system* NEAR/3 (intelligen* or smart*)))

37: (TI =((Artificial* or intelligen*) NEAR/2 (comput* or technol* or network* or imaging or digital*))) OR (AB =((Artificial* or intelligen*) NEAR/2 (comput* or technol* or network* or imaging or digital*))) OR (AK =((Artificial* or intelligen*) NEAR/2 (comput* or technol* or network* or imaging or digital*)))

38: (TI =Generative adversarial network) OR (AB =Generative adversarial network) OR (AK =Generative adversarial network)

39: (TI =(Algorith* NEAR/5 (intelligen* or artificial* or smart* or automat*))) OR (AB =(Algorith* NEAR/5 (intelligen* or artificial* or smart* or automat*))) OR (AK =(Algorith* NEAR/5 (intelligen* or artificial* or smart* or automat*)))

40: (TI =(Intelligen* NEAR/2 (analys* or analyz* or system* or screen* or informatic* or application* or app or apps or process* or simulation* or interactive* or interaction*))) OR (AB =(Intelligen* NEAR/2 (analys* or analyz* or system* or screen* or informatic* or application* or app or apps or process* or simulation* or interactive* or interaction*))) OR (AK =(Intelligen* NEAR/2 (analys* or analyz* or system* or screen* or informatic* or application* or app or apps or process* or simulation* or interactive* or interaction*)))

41: (TI = (("decision tree*" or "random forest*" or "knowledge representation*" or "computer vision system*" or "computer reasoning*" or "natural language processing*" or "connectionist model*") NEAR/2 artificial*)) OR (AB = (("decision tree*" or "random forest*" or "knowledge representation*" or "computer vision system*" or "computer reasoning*" or "natural language processing*" or "connectionist model*") NEAR/2 artificial*)) OR (AK = (("decision tree*" or "random forest*" or "knowledge representation*" or "computer vision system*" or "computer reasoning*" or "natural language processing*" or "connectionist model*") NEAR/2 artificial*))

42: (TI =(AI NOT (aromatase inhibitor* or anatomic insertion* or AI-IgG or apnea index or anti-infective or angiogenesis inhibitors))) OR (AB =(AI NOT (aromatase inhibitor* or anatomic insertion* or AI-IgG or apnea index or anti-infective or angiogenesis inhibitors))) OR (AK =(AI NOT (aromatase inhibitor* or anatomic insertion* or AI-IgG or apnea index or anti-infective or angiogenesis inhibitors)))

43: (TI =(machinelearn* or machine-learn*)) OR (AB =(machinelearn* or machine-learn*)) OR (AK =(machinelearn* or machine-learn*))

44: (TI =(neural network* NEAR/1 (artificial* or convolutional or deep*))) OR (AB =(neural network* NEAR/1 (artificial* or convolutional or deep*))) OR (AK =(neural network* NEAR/1 (artificial* or convolutional or deep*)))

45: (TI=((assist* or aid* or diagnos* or screen*) NEAR/2 (intelligen* or artificial* or smart* or automat*))) OR (AB=((assist* or aid* or diagnos* or screen*) NEAR/2 (intelligen* or artificial* or smart* or automat*))) OR (AK=((assist* or aid* or diagnos* or screen*) NEAR/2 (intelligen* or artificial* or smart* or automat*)))

46: #34 OR #35 OR #36 OR #37 OR #38 OR #39 OR #40 OR #41 OR #42 OR #43 OR #44 OR #45

47: (TI=(telemedicine* or tele-medicine* or remote medicine*)) OR (AB=(telemedicine* or tele-medicine* or remote medicine*)) OR (AK=(telemedicine* or tele-medicine* or remote medicine*))

48: (TI=(tele-health* or telehealth* or remote health*)) OR (AB=(tele-health* or telehealth* or remote health*)) OR (AK=(tele-health* or telehealth* or remote health*))

49: (TI=(telecare* or tele-care* or remote care)) OR (AB=(telecare* or tele-care* or remote care)) OR (AK=(telecare* or tele-care* or remote care))

50: (TI=((remote* or tele*) NEAR/2 ("patient monitor*" or consult* or deliver* or intervention* or treatment* or diagnos* or service* or program* or prevent*))) OR (AB=((remote* or tele*) NEAR/2 ("patient monitor*" or consult* or deliver* or intervention* or treatment* or diagnos* or service* or program* or prevent*))) OR (AK=((remote* or tele*) NEAR/2 ("patient monitor*" or consult* or deliver* or intervention* or treatment* or diagnos* or service* or program* or prevent*)))

51: (TI=(videoconferenc* or video-conferenc* or videoconsult* or video-consult*)) OR (AB=(videoconferenc* or video-conferenc* or videoconsult* or video-consult*)) OR (AK=(videoconferenc* or video-conferenc* or videoconsult* or video-consult*))

52: #47 OR #48 OR #49 OR #50 OR #51

53: (TI=(phone* or telephone* or smartphone* or cellphone* or cell-phone* or smartwatch*)) OR (AB=(phone* or telephone* or smartphone* or cellphone* or cell-phone* or smartwatch*)) OR (AK=(phone* or telephone* or smartphone* or cellphone* or cell-phone* or smartwatch*))

54: (TI=((phone* or telephone* or smartphone* or cellphone* or smartwatch*) NEAR/3 (based or application* or intervention* or program* or therap*))) OR (AB=((phone* or telephone* or smartphone* or cellphone* or smartwatch*) NEAR/3 (based or application* or intervention* or program* or therap*))) OR (AK=((phone* or telephone* or smartphone* or cellphone* or smartwatch*) NEAR/3 (based or application* or intervention* or program* or therap*)))

55: (TI=(Smartphone* or smart-phone*)) OR (AB=(Smartphone* or smart-phone*)) OR (AK=(Smartphone* or smart-phone*))

56: (TI=(Cell-phone* or cellphone*)) OR (AB=(Cell-phone* or cellphone*)) OR (AK=(Cell-phone* or cellphone*))

57: (TI=Mobile-phone*) OR (AB=Mobile-phone*) OR (AK=Mobile-phone*)

58: (TI=((text* or txt) NEAR/3 message*)) OR (AB=((text* or txt) NEAR/3 message*)) OR (AK=((text* or txt) NEAR/3 message*))

59: (TI=(SMS or MMS)) OR (AB=(SMS or MMS)) OR (AK=(SMS or MMS))

60: (TI=mhealth) OR (AB=mhealth) OR (AK=mhealth)

61: (TI=mobile comput*) OR (AB=mobile comput*) OR (AK=mobile comput*)

62: (TI=(m-health or mhealth or m-therapy or mtherapy or mobile health*)) OR (AB=(m-health or mhealth or m-therapy or mtherapy or mobile health*)) OR (AK=(m-health or mhealth or m-therapy or mtherapy or mobile health*))

63: (TI=((mobile* or internet*) NEAR/2 intervention*)) OR (AB=((mobile* or internet*) NEAR/2 intervention*)) OR (AK=((mobile* or internet*) NEAR/2 intervention*))

64: (TI=(mobile health or mhealth or m-health or ehealth or e-health or emental or e-mental)) OR (AB=(mobile health or mhealth or m-health or ehealth or e-health or emental or e-mental)) OR (AK=(mobile health or mhealth or m-health or ehealth or e-health or emental or e-mental))

65: (TI=(("mobile health" or mhealth or m-health or ehealth or e-health or emental or e-mental) NEAR/3 (based or application* or intervention* or program* or therap*))) OR (AB=(("mobile health" or mhealth or m-health or ehealth or e-health or emental or e-mental) NEAR/3 (based or application* or intervention* or program* or therap*))) OR (AK=(("mobile health" or mhealth or m-health or ehealth or e-health or emental or e-mental) NEAR/3 (based or application* or intervention* or program* or therap*)))

66: (TI=(mobile* NEAR/3 (based or application* or intervention* or device* or technolog*))) OR (AB=(mobile* NEAR/3 (based or application* or intervention* or device* or technolog*))) OR (AK=(mobile* NEAR/3 (based or application* or intervention* or device* or technolog*)))

67: (TI=(mobile* NEAR/3 (based or application* or intervention* or device* or technolog*))) OR (AB=(mobile* NEAR/3 (based or application* or intervention* or device* or technolog*))) OR (AK=(mobile* NEAR/3 (based or application* or intervention* or device* or technolog*)))

68: (TI=((mhealth or m-health or ehealth or e-health or "technology enabled care" or "digital health*" or "digital medicine*" or eportal* or e-portal* or "digital portal*" or "virtual health*" or "mobile health*" or "mobile intervention*" or "mobile comput*" or "Internet of Things" or IoT or epatient* or e-patient* or online or web* or internet or zoom) NEAR/3 (service* or program* or intervention* or deliver* or remote* or treatment* or diagnos* or prevent* or therap* or setting* or session* or remote* or train* or technolog*))) OR (AB=((mhealth or m-health or ehealth or e-health or "technology enabled care" or "digital health*" or "digital medicine*" or eportal* or e-portal* or "digital portal*" or "virtual health*" or "mobile health*" or "mobile intervention*" or "mobile comput*" or "Internet of Things" or IoT or epatient* or e-patient* or online or web* or internet or zoom) NEAR/3 (service* or program* or intervention* or deliver* or remote* or treatment* or diagnos* or prevent* or therap* or setting* or session* or remote* or train* or technolog*))) OR (AK=((mhealth or m-health or ehealth or e-health or "technology enabled care" or "digital health*" or "digital medicine*" or eportal* or e-portal* or "digital portal*" or "virtual health*" or "mobile health*" or "mobile intervention*" or "mobile comput*" or "Internet of Things" or IoT or epatient* or e-patient* or online or web* or internet or zoom) NEAR/3 (service* or program* or intervention* or deliver* or remote* or treatment* or diagnos* or prevent* or therap* or setting* or session* or remote* or train* or technolog*)))

69: (TI=mobile health*) OR (AB=mobile health*) OR (AK=mobile health*)

70: (TI=mobile intervention*) OR (AB=mobile intervention*) OR (AK=mobile intervention*)

71: (TI=(mobile NEAR/3 (comput* or device* or application* or technolog* or platform* or software*))) OR (AB=(mobile NEAR/3 (comput* or device* or application* or technolog* or platform* or software*))) OR (AK=(mobile NEAR/3 (comput* or device* or application* or technolog* or platform* or software*)))

72: (TI=((App or Apps) not (mutant or mice or amyloid precursor protein* or gene dose* or acute phase protein*))) OR (AB=((App or Apps) not (mutant or mice or amyloid precursor protein* or gene dose* or acute phase protein*))) OR (AK=((App or Apps) not (mutant or mice or amyloid precursor protein* or gene dose* or acute phase protein*)))

73: (TI=(tablet* NEAR/3 (application* or device* or technolog* or platform* or mobile* or comput* or software*))) OR (AB=(tablet* NEAR/3 (application* or device* or technolog* or platform* or mobile* or comput* or software*))) OR (AK=(tablet* NEAR/3 (application* or device* or technolog* or platform* or mobile* or comput* or software*)))

74: (TI=(mobile application* or Appstore or App-based)) OR (AB=(mobile application* or Appstore or App-based)) OR (AK=(mobile application* or Appstore or App-based))

75: #53 OR #54 OR #55 OR #56 OR #57 OR #58 OR #59 OR #60 OR #61 OR #62 OR #63 OR #64 OR #65 OR #66 OR #67 OR #68 OR #69 OR #70 OR #71 OR #72 OR #73 OR #74

76: (TI=wearable*) OR (AB=wearable*) OR (AK=wearable*)

77: #76 OR #75 OR #52 OR #46 OR #33 OR #30

78: #77 AND #4

## Web of Science (preprint)

1: (TI="target product profil*") OR (AB="target product profil*")

2: (TI="QTPP") OR (AB="QTPP")

3: (TI="quality by design") OR (AB="quality by design")

4: #1 OR #2 OR #3

## ACM Digital Library

Title:("quality by design") OR Abstract:("quality by design") OR Title:("target product profil*") OR Abstract:("target product profil*") OR Title:(QTPP?) OR Abstract:(QTPP?)

## Web search

1. Google searches (signed out, browser cookies cleared) performed independently by two reviewers (TM, LV) on 22/06/2023 and 08/07/2023 respectively. 100 hits for each combination of the search terms below screened for relevance. A maximum of three clicks/pages from the hit was permitted to decide whether a hit was relevant, otherwise this was passed on to title/abstract review.

- "Target product profile" AND "artificial intelligence"
- "Target product profile" AND digital
- "Target product profile" AND telemedicine
- "Target product profile" AND app
- "Target product profile" AND smartphone
- "Target product profile" AND wearable
- TPP AND AI
- TPP AND "artificial intelligence"
- TPP AND digital
- TPP AND telemedicine
- TPP AND app
- TPP AND smartphone
- TPP AND wearable
- QTPP AND AI
- QTPP AND artificial intelligence
- QTPP AND digital
- QTPP AND telemedicine
- QTPP AND app
- QTPP AND smartphone
- QTPP AND wearable

1. Individual websites searched through native search bars using the term "target product profile". Relevant websites were taken from Cocco et al. (2020) or identified from the Google searches above. If websites had no search function Google was used to search the website specifically e.g. site:www.finddx.org 'target product profile'.

| **Name** | **Dates searched** | **URL** | **Source** |
| --- | --- | --- | --- |
| AIGHD | 23/06/2023 & 18/07/2023 | https://www.aighd.org/ | Cocco et al. |
| CanTest | 23/06/2023 & 18/07/2023 | https://www.cantest.org/ | Google search |
| CF AMR Syndicate | 23/06/2023 & 18/07/2023 | https://cfamr.org.uk/ | Google search |
| Concept Foundation | 23/06/2023 & 18/07/2023 | https://www.conceptfoundation.org/ | Google search |
| Critical Path Institute | 23/06/2023 & 18/07/2023 | https://c-path.org/ | Cocco et al. |
| DiaDev (Investigating diagnostics in global health) | 23/06/2023 & 18/07/2023 | http://www.diadev.eu/ | Cocco et al. |
| DNDi ( Drugs for Neglected Diseases initiative) | 23/06/2023 & 18/07/2023 | https://www.dndi.org/ | Cocco et al. + Google search |
| EMA | 23/06/2023 & 18/07/2023 | https://www.ema.europa.eu/ | Cocco et al. + Google search |
| European Directorate for the Quality of Medicines & HealthCare | 23/06/2023 & 18/07/2023 | https://www.edqm.eu/ | Cocco et al. |
| FDA | 23/06/2023 & 18/07/2023 | https://www.fda.gov | Cocco et al. + Google search |
| FIND | 23/06/2023 & 18/07/2023 | https://www.finddx.org | Cocco et al. + Google search |
| Galvmed | 23/06/2023 & 18/07/2023 | https://www.galvmed.org | Cocco et al. |
| Government | 23/06/2023 & 18/07/2023 | www.gov.uk | Google search |
| United Nations Global Marketplace | 23/06/2023 & 18/07/2023 | https://www.ungm.org/ | Google search |
| ICH harmonisation for better health | 23/06/2023 & 18/07/2023 | https://www.ich.org | Cocco et al. |
| IMPT for Reproductive Health | 23/06/2023 & 18/07/2023 | https://www.theimpt.org/ | Cocco et al. |
| IVCC | 23/06/2023 & 18/07/2023 | http://www.ivcc.com/ | Cocco et al. + Google search |
| Long-Acting/Extended Release Antiretroviral Research Resource Program | 23/06/2023 & 18/07/2023 | https://leapresources.org/ | Google search |
| Malaria consortium | 23/06/2023 & 18/07/2023 | https://www.malariaconsortium.org/ | Cocco et al. |
| Medicines for Malaria Venture | 23/06/2023 & 18/07/2023 | https://www.mmv.org/research-development/information-scientists/target-product-profiles-target-candidate-profiles | Google search |
| NICE | 23/06/2023 & 18/07/2023 | https://www.nice.org.uk/ | Cocco et al. |
| PATH | 23/06/2023 & 18/07/2023 | https://path.org | Cocco et al. + Google search |
| Pink Ribbon | 23/06/2023 & 18/07/2023 | http://pinkribbonredribbon.org/ | Cocco et al. |
| Product Quality Research Institute | 23/06/2023 & 18/07/2023 | http://pqri.org/ | Cocco et al. |
| Public Health Emergency | 23/06/2023 & 18/07/2023 | https://www.phe.gov/ | Cocco et al. |
| ReAct group | 23/06/2023 & 18/07/2023 | https://www.reactgroup.org/ | Cocco et al. |
| STP TB Partnership | 23/06/2023 & 18/07/2023 | http://www.stoptb.org | Cocco et al. |
| The International Diagnostics centre London School of Hygiene & Tropical Medicine | 23/06/2023 & 18/07/2023 | http://www.idc-dx.net | Cocco et al. |
| UNICEF | 23/06/2023 & 18/07/2023 | https://www.unicef.org | Cocco et al. + Google search |
| Unitaid | 23/06/2023 & 18/07/2023 | https://unitaid.org | Cocco et al. |
| Wellcome | 23/06/2023 & 18/07/2023 | https://wellcome.org/ | Google search |
| WHO | 23/06/2023 & 18/07/2023 | http://www.who.int | Cocco et al. + Google search |
